# Recent trends in pulmonary arterial hypertension

**DOI:** 10.4103/0970-2113.76300

**Published:** 2011

**Authors:** Rajagopalan Natarajan

**Affiliations:** *Department of Pulmonary and Critical Care Medicine, University of Massachusetts, Worcester, MA, USA*

**Keywords:** Idiopathic pulmonary hypertension, primary pulmonary hypertension pulmonary arterial hypertension, pulmonary hypertension, pulmonary vascular diseases, secondary pulmonary hypertension

## Abstract

Pulmonary hypertension is a serious and unrelenting pulmonary vascular disorder that affects the functional quality of patients and significantly decreases their life span. If diagnosed early, with the number of new therapeutic options that are available, a better quality of life can be provided for a protracted length of time. It is likely that the available treatment will change the natural course of the disease and perhaps prolong survival. As symptoms are often subtle in the early stages of the disease it is imperative that physicians are aware of the manifestations of this condition. A thorough investigation of patients suspected of this condition is essential so that appropriate treatment can be initiated promptly. The routine workup of a patient suspected to have pulmonary hypertension could easily be carried out in any well-equipped peripheral hospital in many affluent and advanced countries. However, it must be mentioned that in some less advanced countries the necessary work up can only be done in major teaching hospitals. Both pulmonologists and cardiologists should be aware of the pathophysiology of pulmonary arterial hypertension, the workup and the treatment options that are available. Patients with refractory pulmonary hypertension should be referred to these research centers for enrolment into any ongoing drug trials as well as for evaluation for heart–lung, single lung, or double lung transplantation. This paper is primarily aimed at pulmonologists and cardiologists taking care of these patients. Unless indicated otherwise this paper mainly deals with WHO group 1 pulmonary hypertension which is designated pulmonary arterial hypertension. Extensive review of the literature spanning the last 30 years was made through Medline using titles such as primary pulmonary hypertension, pulmonary arterial hypertension, secondary pulmonary hypertension, and pulmonary vascular diseases.

## INTRODUCTION

A couple of decades ago pulmonary hypertension (PH) was considered a rapidly fatal illness with a median survival of only 2.8 years. Significant advances have been made in this field in the last decade both in the understanding of the pathophysiology and the treatment of PH. In the past, even though physicians were able to make a diagnosis of PH, treatment of this condition was often unsuccessful and patients invariably succumbed to the disease. The discovery of prostacyclin by Sir John Vane made a revolution in the treatment of PH. In the past, PH was considered either primary or secondary. It soon became clear that this was an over simplification. PH from a number of unrelated conditions was grouped as secondary pulmonary hypertension. Hence, treatment of PH in these conditions could not be evaluated meaningfully. The newer WHO classification is very helpful in this regard.

Until recently, the diagnosis of pulmonary hypertension was based upon a mean pulmonary arterial pressure of greater than 20 mm mercury. However, the upper limit of normal mean pulmonary artery pressure is now generally agreed to be 25 mm mercury.[1] For the diagnosis of pulmonary hypertension it is also required that the mean pulmonary capillary wedge pressure and left ventricular end diastolic pressure must be less than 15 mm mercury, and a pulmonary vascular resistance of >3 Wood units.[1] A few years ago it was felt that earlier on in the disease, patients may have normal pulmonary pressure at rest and with exercise would show an elevated pulmonary arterial pressure. It was also felt that these patients might develop a full-blown pulmonary arterial hypertension with time. It is now felt that these patients merely show a hypertensive response to exercise just like what is seen in systemic vasculature and they do not necessarily develop a full-blown disease.

## CLASSIFICATION, ETIOLOGY, AND PATHOGENESIS

On the recommendation of experts in the field of pulmonary artery hypertension, WHO adopted a classification based on the pathophysiology into five different groups.[2]

Group I consists of Idiopathic and familial pulmonary hypertension. This group is also designated The Pulmonary artery Hypertension group. Group II includes left-sided valvular diseases and recently recognized diastolic dysfunction related PAH. Group III consists of interstitial lung diseases, chronic obstructive pulmonary disease, and obstructive sleep apnea-related pulmonary arterial hypertension (PAH). Group IV includes pulmonary thrombo embolic diseases. Group V includes miscellaneous diseases such as sarcoidosis, etc.

The etiology of idiopathic pulmonary hypertension is still unclear. A definite association of PAH and medications has been recognized with the appetite suppressants such as aminorex,[3,4] fenfluramine, and dexfenfluramine,[5] as well as tryptophan in the setting eosinophlia-myalgia syndrome.[6] An epidemic of PH was noted in Spain in the last century in people ingesting rapeseed oil contaminated with aniline and acetanilide dyes.[7]

Among connective tissue diseases, pulmonary arterial hypertension has been recognized frequently in association with a variant of scleroderma called Calcinosis, Reynaud’s phenomenon, esophageal dysmotility, and telangiectasia (CREST) syndrome. At autopsy, 80% of patients with CREST syndrome have histopathological changes of PAH. However, in only 15% of the patients, was the disease recognized premortem.[8–10] Pulmonary artery hypertension has also been recognized in association with other connective tissue diseases such as systemic lupus erythematosis (SLE), rheumatoid arthritis, and mixed connective tissue diseases. It is frequently associated with Reynaud’s phenomena indicating similar pathogenesis in these diseases.[11]

An association between human immunodeficiency virus (HIV) infection and PAH was noted in 1991. Initial cases occurred primarily in hemophiliacs who acquired HIV infection after receiving factor VIII enriched plasma.[12,13] PAH has now been recognized in patients with HIV infection acquired through different routes. The incidence of PAH in HIV patients has been found to be 0.5%, which is about six times as high as in general population. The occurrence of PAH is related to the length of HIV infection, presence of Hepatitis B or C and presence of foreign body emboli in the pulmonary circulation secondary to IV drug abuse. The exact mechanism of PAH in HIV infection is unclear and it must be mentioned that HIV does not affect endothelial cells. Human herpes virus 8 (HHV-8), the causative agent of Kaposi’s sarcoma has also been strongly associated with PAH.[14]

Genetic factors in pulmonary arterial hypertension have received great attention. Mutations in bone morphogenetic protein receptor 2(BNP2) gene have been noted in 70% of cases with a family history.[15] They have also been noted in 11%–40% of apparently idiopathic cases without any familial history. It is believed that these are instances of de novo mutation, hence the term familial is no longer used and is replaced by the term heritable. So far 298 mutations have been described involving BMPR2 gene. Most of these mutations are unique to the family affected by PAH. PAH has been noted in approximately 6% of the patients referred for liver transplantation. All of these patients have portal hypertension. The risk of PAH is significantly higher, the longer the duration of portal hypertension.[16,17]

Several studies have documented pulmonary arterial hypertension and right ventricular dysfunction in hemoglobinopathies. The incidence of PAH has been estimated to be 8%–30%.[18] The destruction of nitric oxide by free hemoglobin and increased production of reactive oxygen species seem important in the development of PAH.[19–21]

Pulmonary arterial hypertension has been noted in about 15% of cases of hereditary hemorrhagic telangiectasia. Mutations involving transforming growth factors (TGF) beta receptors namely endoglin and activin receptor like kinase (ALK 1) have been associated with PAH in this condition.[22]

## INCIDENCE

Estimates of the incidence of pulmonary arterial hypertension range from one to two cases per million in the general population.[23] The incidence of PAH in portal hypertension or in HIV infection has been estimated to be between 0.5% and 2%.[12,24]

The familial form of pulmonary arterial hypertension does not differ from the sporadic form of the disease in the affected individual. However, it is associated with a pattern of “genetic anticipation,” a worsening of disease in subsequent generations manifested by severity or early onset.[25]

## PATHOLOGY AND PATHOGENESIS

The pulmonary vasculature in pulmonary arterial hypertension belonging to group 1 WHO classification share certain common pathological findings. They include muscularization of non-muscularized distal pulmonary arterioles, formation of neo- intima by recruitment of myofibroblasts with deposition of extracellular mattix between the endothelium and internal elastic lamina. The plexiform lesions considered to be characteristic of PAH result from development of new endothelial channels within the intima of the affected blood vessels. These endothelial cells show monoclonality and resistance to apoptosis.

A number of vasoactive substances are involved in the pathogenesis of PAH. A major imbalance between prostacyclin and thromboxane has been noted in PAH. Both of them are products of arachidonic acid metabolism by the endothelial cells. Prostacyclin increases intracellular cyclic AMP and they by reduces intracellular calcium.

Transcription factors and cell cycle progression are dependent on calcium. Prostacyclin inhibits platelet activation, promote vasodilatation and inhibits smooth muscle proliferation. Thromboxane, which is also produced by the endothelium antagonizes the effects of prostacyclin. The imbalance between prostacyclin and thromboxane may result from a combination of genetic factors and aberrant response to some kind of injury to the endothelial cells. Endothelin-1 is produced by vascular endothelium and it serves as a vasoconstrictor and smooth muscle mitogen. While its action on endothelin-A receptors results in vasoconstriction through activation of protein kinase and increase in intracellular calcium, its effect on endothelin-B receptors results in vasodilatation secondary to prostacyclin and nitric oxide release and also aids in its own clearance. Nitric oxide is produced by the action of nitric oxide synthase on L-arginine in the pulmonary vascular smooth muscle cells. NO increases cyclic GMP by activating guanylate cyclase, which in turn activates potassium channels. This results in inactivation of calcium channel activity and decreases the intracellular availability of calcium causing vasodilatation. Patients with PAH show impaired NO synthase activity. Impaired synthesis of endothelium-derived nitric oxide and enhanced production of vasoconstrictor endothelin have also been implicated in the pathogenesis of PAH.[26,27] Serotonin (5-HTT) has been implicated in the pathogenesis of PAH. Serotonin inhibits cyclic AMP and thereby causes vasoconstriction. A number of transporters have been described that are involved in the transportation of serotonin across the cell membranes. Patient with PAH have a higher frequency of the *L. allelic* variant of 5-HTT gene promoter leading to increased production of serotonin.[28]

Vasoactive intestinal peptide (VIP) has been shown to reduce the pulmonary arterial pressure in rabbit with monocrotaline-induced pulmonary arterial hypertension[29] and also in healthy human subjects.[30] Decreased levels of VIP in serum and lung have been noted in patients with PAH and treatment with inhaled VIP has also been shown to improve the hemodynamics and clinical course in these patients.[31]

Production of vascular endothelial growth factor (VEGF) and its receptors VEGF receptor-1 and VEGF receptor-2 is increased in patients with pulmonary arterial hypertension.[32]

Mutations in the gene encoding bone morphogenetic protein receptor II (BNPR2) have been found in families with PAH.[33] These act as ligands in the growth factor beta family. Activation of these receptors leads to signaling via a Smad proteins and other signaling pathways leaving to gene transcription. Interestingly only 20% of members manifesting this mutation manifest PAH in the familial form.

## DIAGNOSIS AND ASSESSMENT OF PULMONARY ARTERIAL HYPERTENSION

Clinical assessment for pulmonary arterial hypertension is often difficult and insensitive. Typical findings of pulmonary arterial hypertension such as an accentuated P2, right ventricular thrust and signs of right ventricular failure are very late manifestations in the evolution of a full-blown pulmonary arterial hypertension. Initial evaluation in a patient suspected to have pulmonary arterial hypertension will consist of an electrocardiogram looking for P pulmonale and evidence of right ventricular hypertrophy and strain pattern. Chest X-ray may show enlarged pulmonary arteries and obliteration of retrosternal space in the lateral chest X-ray which can very easily be missed if not looked for specifically. CT scan of the chest with contrast is essential to rule out pulmonary thromboembolic disease. In pulmonary hypertension the enlarged pulmonary arteries and the right ventricle can be better appreciated in the CT scan of the chest than in the chest X-ray. In chronic thromboembolic disease ventilation perfusion scan is more sensitive in showing a Mosaic pattern typical for this condition. While the above-mentioned tests are considered routine, the most essential investigation in the diagnosis and evaluation of pulmonary arterial hypertension is transthoracic echocardiogram.

Pulmonary arterial systolic pressure can be determined by measuring the peak systolic pressure gradient from the right ventricle to right atrium. This is calculated by a modified Bernoulli equation. The formula that is used is 4*V*^2^, where *V* is the maximum velocity of the tricuspid regurgitant jet measured by continuous wave Doppler. This is added to the right atrial pressure. A commonly used method to measure the right atrial pressure is to determine the variation in the size of the inferior vena cava with inspiration. Complete collapse of the IVC indicates a right atrial pressure of 5 mm mercury, partial collapse indicates 10 mm mercury pressure and absence of collapse indicates more than 15 mm mercury.[34,35] The problem with estimation of pulmonary arterial pressure based on tricuspid regurgitant jet is that in only 80% of patients with pulmonary artery pressure of greater than 35 mm mercury is the tricuspid regurgitant jet detected. This increases to 95% when the pulmonary arterial pressure is greater than 50 mm mercury. Finally, it must be mentioned that by echocardiography only an estimate of peak pulmonary systolic pressure can be made while a diagnosis of pulmonary hypertension is based on mean pulmonary artery pressure. However, there seems to be a good correlation between ECHO determined estimated pulmonary artery pressure and mean pulmonary pressure.

In addition, the presence of analyzable tricuspid regurgitant jet seems to depend on the body habits and the type of underlying lung disease. Estimates of pulmonary arterial systolic pressure in patients with advanced lung disease such as fibrosis and COPD could be very difficult. In a cohort of 400 patients, only 166 (44%) had adequate image and acoustic window to allow estimates of pulmonary artery systolic pressure.[36]

Transthoracic echocardiogram has a lot of intrinsic limitations and operator dependency. Doppler echo can overestimate PASP by more than 10 mm mercury in 50% of patients without PAH and in 30% of patients with PAH.[35] It is generally believed that tricuspid jet velocity (TJV) strongly correlates with pulmonary systolic pressure and directly measured pulmonary systolic pressure correlates well with mean pulmonary artery pressure. TJV greater than 2.8 m/s and tricuspid insufficiency pressure gradient (TIPG) greater than 31 mm mercury are diagnostic of pulmonary arterial hypertension. A new echocardiographic measurement of tricuspid annular plane systolic excursion (TAPASE) toward the apex has a prognostic value. Right ventricular outflow tract velocity profiles can be generated using measurement of pulmonary flow proximal to the pulmonary valve in the middle of the outflow tract.[37] Several pulmonary flow indices such as pre-ejection period, acceleration time, and right ventricular ejection time can be calculated based on these velocity profiles. Acceleration time varies inversely with increasing pulmonary vascular resistance and this may be a more useful measurement than estimation of mean PAP. Additional measurements of RV systolic function can be done with tissue Doppler imaging (TDI) of tricuspid annular velocity or RV index of myocardial performance (Tei). Velocity less than 10 cm/s reflect abnormal RV function. RV ejection time can be estimated with pulsed wave Doppler (PW) in the RV outflow tract.

Even though right heart catheterization has been considered as the gold standard in the assessment of pulmonary arterial hypertension, cardiac MRI may soon supersede this as the gold standard.[38,39] MRI demonstrates good inter study reproducibility for RV function in healthy subjects, patients with right ventricular hypertrophy and right heart failure. RV mass which is difficult to quantify by other methods can be accurately done by MRI.[40] By MRI, it can be shown that right ventricle has a crescent shape and left ventricle has a more circular shape. When the right ventricle pressure is elevated quite the opposite happens. This can be well demonstrated by MRI. Right ventricular function appears to be the most important determinant of life expectancy in patients with PAH.[41] For the time being MRI is considered a more experimental tool in the diagnosis and follow up of patient’s with PAH.

Lung biopsy is reserved only in an unusual patient in whom PH is not thought to be the primary disease. In such patients, one always finds abnormalities such as lung infiltrates or other findings pointing to other etiologies such as pulmonary hemangiomatosis or pulmonary venoocclusive disease.

Even though there are no clearly defined parameters or biomarkers that can be relied upon, measurement of NT-proBNP in the serum and measurement of tricuspid annular plane systolic excursion (TAPSE) toward the apex which can be used as a surrogate for RV systolic function may prove to be reliable markers. These in addition to 6 min walk distance could provide the prognostic information necessary in following up these patients. Maintaining patients in a lower functional class is essential in the management of PAH. Treatment may have to be escalated if no improvement is seen with regard to the functional class after 3 months of treatment. All the landmarks trials have shown poor prognosis when patient remain in functional class III or IV after 3 months of treatment.

## TREATMENT ALGORITHM IN PAH

Treatment of PAH consists of general and specific measures. General measures include oxygen, diuretics, and restriction of physical activity, etc. Specific measures include medications that are mainly targeted for PAH.

**Figure 1:**
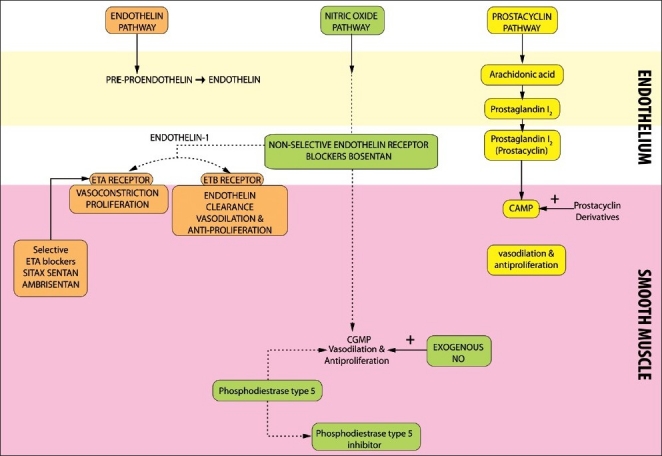
The three major pathways involved in regulation of vasomotor tone in the pulmonary vasculature

Intravenous epoprostenol was introduced in 1995. Since then significant advances in the treatment of PAH have been made in the last 15 years, with eight new medical therapies having been approved. These agents target the prostacyclin pathway, nitric oxide pathway and the endothelin pathway [Figure 1]. In addition, combination trials have clearly demonstrated synergistic benefit by targeting two or three pathways.

### Nitric oxide

Nitric oxide treatment is very cumbersome and its only possible role is in the management of pulmonary hypertension in neonatal ICU and occasionally in adult ICU. Even though anecdotal reports of improvement in oxygenation have been reported in ARDS patients, outcome measures such as mortality have not been shown to be favorably affected by nitric oxide treatment.

### Calcium channel blockers

High-dose calcium channel blockers have been shown by uncontrolled studies to prolong survival in patients with pulmonary arterial hypertension.[42–44] About 10% of patients with PAH belong to this group. Interestingly, patients with BMPR2 receptor mutation do not respond to calcium channel blockers. Signaling through BMPR2 and other similar receptors plays an important role in intimal and smooth cell proliferation and has no role in maintenance of vasomotor tone. Patient who may benefit from calcium channel blocker therapy can be identified by performing an acute vasodilator response test using inhaled nitric oxide, intravenous prostacyclin or adenosine during right heart catheterization.[43,44] The magnitude of the short-term response to vasodilator that predicts long-term response to calcium channel blockers is not well defined.[43,44] It is widely accepted that 20% reduction in mean pulmonary arterial pressure and or pulmonary vascular resistance is essential to undertake therapy with calcium channel blockers. In a large retrospective study of 557 patients with pulmonary arterial hypertension, less than 7% of patients responded to calcium channel blockers. Those patients who had a significant vasodilator response had a long-term response to calcium channel blockers. Long-term therapy with calcium channel blockers is not recommended when there is no acute vasodilator response.[44]

### Warfarin

The role of warfarin in the treatment of pulmonary arterial hypertension is not well defined. However, a large number of patients dying with PAH postmortem studies have shown a high incidence of thrombosis.[44] Warfarin has been evaluated in two studies, one retrospective and one prospective. The authors had recommended keeping the INR between 1.5 and 2. Even though there are no double blind placebo controlled trials warfain is recommended by both AHA and ACP in functional class III and IV patients with idiopathic pulmonary hypertension.

### Prostacyclin therapy

Prostacyclin, the main product of arachidonic acid in the vascular endothelium causes relaxation of smooth muscle by stimulating the production of cyclic AMP and also results in inhibition of growth of smooth muscle cells.[45] Intravenous prostacyclin was first introduced in the treatment of primary pulmonary arterial hypertension in the early 1980s. Forty-four Cohort analysis of patients receiving intravenous prostacyclin has shown benefits in NYHA class III and IV with regard to survival.[46,47]

In addition to idiopathic pulmonary arterial hypertension, epoprostenol has been successfully used in the treatment of pulmonary hypertension resulting from left to right shunt, portal hypertension and HIV infection.[48–51]

The normal dose of epoprostenol is 21 ng/kg/min. The dose can be increased up to 32 ng/kg/ min after a year. Epoprostenol has a definite role in the treatment of primary and other forms of pulmonary hypertension. However, it is expensive and very cumbersome to use. Administration of this medication requires a central line and a pump. Central line infection is a serious side effect. Other side effects include flushing, headache, jaw pain, leg pain, nausea, and diarrhea. Care should be taken when using this medication in venoocclusive disease and pulmonary capillary hemangiomatosis. Severe pulmonary edema has been reported with use of epoprostenol in these conditions. The reason for this is because of increased perfusion in the presence of vascular obstruction downstream.[52,53]

### Treprostinil

Treprostinil is a stable prostacyclin that can be administered either subcutaneously or intravenously. The usual dose is 0.625–40 ng/kg/min.[54,55] When switching from epoprostenol the starting dose of treprostinil is usually 10% of the dose of epoprostenol the patient was receiving at the time of switch. Side effects are the same as that of epoprostenol.

Treprostinil can also be given as an inhalation usually in the form of a nebulizer. The usual dose is 18 mcg (three breaths) four times a day. The dosage is usually increased to 54 mcg (nine breaths) four times a day in a few weeks. Common side effects of inhaled treprostinil include epistaxis, hemoptysis, hypotension, and syncope in addition to other side effects that are common to epoprostenol.

Beraprost sodium is a prostacyclin analog that has been approved in Japan. Clinical trials have shown improvement in 6-min walk test but not in the hemodynamics. The dose that has been used in Japan is 18 mcg four times a day.[56,57]

### Inhaled iloprost

Iloprost is a stable prostacyclin analog that can be given as an inhaled medication. The optimal particle size is 0.5–3 mcg to ensure alveolar deposition. The usual dose of iloprost is 2.5–5 mcg by nebulizer q.2–4 h.[58–60]

### Endothelin receptor antagonist

#### Bosentan

Bosentan is a nonselective endothelial receptor antagonist. Endothelin A receptor stimulation results in vasoconstriction and smooth muscle proliferation. Endothelin B receptor stimulation results in endothelin clearance and induction of nitric oxide and prostacyclin by endothelial cells. Sitaxsentan and ambrisentan are selective ETA receptor antagonists. Bosentan is metabolized by the liver and an increase in transaminases has been noted during treatment with this medication. Hence, it is mandatory to perform periodic liver function tests in patients taking bosentan. The usual dose of bosentan is 62.5 mg twice a day and this can be increased to 125 mg twice a day.

Two randomized double-blind placebo-controlled trials have evaluated bosentan in patients with pulmonary arterial hypertension. In a pilot study,[61] 33 patients with PAH In functional class III or IV were randomized to receive either placebo or bosentan. Patients in the bosentan arm received 62.5 mg of the medication twice daily. After 4 weeks the dose was increased to 125 mg twice daily for at least 12 weeks. Significant improvement in 6 min walk test and hemodynamics were noted in the bosentan group. Subsequently, a large study consisting of 213 patients in functional class III and IV we randomized to receive either placebo or bosentan. After 4 weeks the treatment group was divided into two groups, one receiving 125 mg twice a day and the other 250 mg twice a day for at least 12 weeks.[62] In addition to the improvement in 6 min walk test and hemodynamics, improvement in the time to clinical worsening such as death, lung transplantation, and hospitalization was noted. No dose response effect with respect to the drugs efficacy could be ascertained.

### Sitaxsentan

Sitaxsentan has 7000-fold higher activity for ETA receptors than ETB receptors. The dose of oral Sitaxsentan is 100 mg daily. Hepatotoxicity has been noted as with bosentan.[63–65]

Ambrisentan is another ETA receptor blocker and is given in a dose of 5 mg daily. This can be increased to 10 mg daily. Again acute hepatitis has been described with the use of this medication.

### Phosphodiesterase-5 inhibitors

Sildenafil is a phosphodiesterase 5 (PDE-5) inhibitor that increases the levels of cyclic GMP in smooth muscle cells and causes vasodilatation. It was initially used in angina pectoris and later for erectile dysfunction. The normal dosage in pulmonary arterial hypertension is 20 mg three times a day. Doses as high as 200 mg per day have been used. However, higher doses have not been found to be anymore effective. Common side effects include flushing, epistaxis, and visual disturbances. More serious reactions such as sudden blindness, myocardial infarction, stroke and sudden cardiac death secondary to ventricular arrhythmias, subarachnoid hemorrhage and retinal hemorrhages have been recognized. Tadalafil is a long acting phosphodiesterase 5 inhibitor, which can be given once a day at a dose of 40 mg.

The major landmark trial involving sildenafil was the sildenafil use in pulmonary arterial hypertension (SUPER) study. Two hundred seventy eight patients in functional class II–IV were randomized to three different doses of sildenafil namely 20, 40, or 80 mg three times daily or to placebo. The study extended for 12 weeks and based on the favorable results FDA approved 20 mg three times daily of sildenafil in the treatment of pulmonary arterial hypertension.[66] PHIRST Trial studied the efficacy and safety of long acting phosphodiesterase-type five inhibitor (PDE 5-I) tadalafil. Patients with PAH were either treatment naïve or were already receiving bosentan. The study extended over a period of 16 weeks and the doses of tadalafil used in the study were 2.5, 10, 20, and 40 mg. Based on the favorable response FDA approved 40 mg once a day of tadalafil.[67]

### Combination therapy

Combination therapy to target more than one or two pathways involved in the vasomotor tone of pulmonary vessels is appealing. Combination therapy has already been born out in the treatment of systemic hypertension. Even though it is appealing it is not entirely clear at this time whether combination therapy is more effective than monotherapy and if it alters the natural course of the disease in pulmonary arterial hypertension.

Combination therapies can be either concomitant therapy or add on therapy. To date all published trials have been done with add-on therapy.

### Prostanoids and ETA receptor antagonist

In the recent multicenter placebo-controlled safety and pilot efficacy trial ((STEP) in combination with Bosentan for the evaluation in pulmonary arterial hypertension) iloprost was added to bosentan in patients with dyspnea (NYHA 3 or 4). Improvement in post inhalation hemodynamics and lengthening of time to clinical worsening were noted. Based on this trial, FDA approved iloprost as an add-on therapy in patients receiving bosentan.[68]

### Prostanoids and PDE 5 inhibitors

Among many trials looking at combination of prostacyclin and PDE 5 inhibitors, the most noteworthy is pulmonary arterial hypertension combination study of epoprostenol and sildenafil (PACES). Improvement in symptoms of shortness of breath and increasing 6 min walk test were noted.[69]

### Endothelin receptor antagonist and PDE 5 inhibitors

The benefits of combination treatment with Endothelin receptor antagonist (ERAs) and PDE-5 inhibitors came from EARLY (endothelin antagonist trial in mildly symptomatic PAH patients) trial, COMPASS-1 (hemodynamic effects of a single dose of sildenafil in symptomatic patients on bosentan treatment for PAH).[70,71]

There is a potential interaction between bosentan and sildenafil as both are metabolized through CYP3A4. Bosentan levels may go up and increase side effects of Bosentan such as postural hypotension. Sildenafil levels have also been shown to fall with the combination treatment but these do not seem to affect the management of pulmonary hypertension as the dose of sildenafil can always be adjusted.

The major debate regarding combination treatment is whether a second medication should be used as an add-on medication or whether it should be started concomitantly from the start. There are advantages and disadvantages to each of these. In the add-on modality, the patient should be followed up very closely and a second medication added when there is failure of improvement based on 6-min walk test, hemodynamics and cardiac MRI. If a similarity can be drawn between cancer treatments and PAH, it can very easily be conceived that combination treatment has an edge over add on treatment.[72] The major disadvantages with combination treatment are cost and risks.

Any discussion of pulmonary arterial hypertension will be incomplete without mentioning the role of lung transplantation. Both single and double lung transplantation have been carried out for a select group of patients with pulmonary arterial hypertension when targeted vasodilator therapy does not produce any improvement, and patients continue to remain in functional class III and IV. Decrease in pulmonary vascular resistance occurs within the first 24–48 h and improvement in right ventricular hemodynamics takes anywhere from a few days to a few weeks after lung transplantation. Heart lung transplantation is mostly reserved for children with Eisenmenger syndrome or in adults with severe right ventricular dysfunction. The benefits of lung transplantation have to be carefully weighed against the known complications of lung transplantation namely long-term immunosuppression and graft rejection. Atrial septostomy is not used any more since the advent of epoprostenol. It is reserved in exceptional cases with deteriorating cardiac index and increasing pulmonary vascular resistance despite epoprostenol.

**Figure 2:**
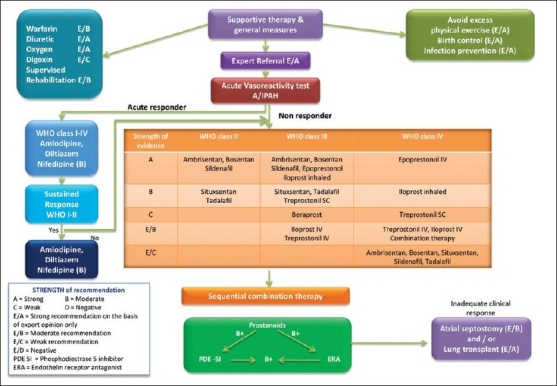
Treatment algorithm for pulmonary arterial hypertension

A step wise algorithm in the management of PAH is illustrated in Figure 2.

### Newer therapies in PAH

Newer agents show a great promise in the treatment of PAH. All the available medications for PAH have some effect on the proliferation of mesenchymal cells namely smooth muscle cells but have no effect on the proliferation of endothelial cells which appears to be the major disturbance in the pathogenesis of PAH.[73]

**Figure 3:**
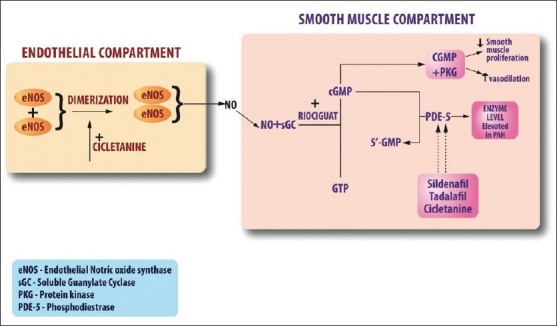
Nitric oxide pathway and the site of action of the new medications cicletanine and riociguat

Newer agents such as cicletanine and riociguat provide much superior effect in comparison with the available agents for PAH. Tyrosine kinase inhibitors may address the neoplastic aspects of PAH and cell based gene therapy to reseed the vascular compartment with normal apoptosis sensitive endothelial cells are certainly in the horizon.[74] Both cyclic guanosine mono phosphate (GMP) and cyclic adenosine mono phosphate (AMP) promote vasodilatation and inhibit smooth muscle proliferation by joining with protein kinase G.[75] The mode of action of the newer drugs is well illustrated in [Figure 3]. In PAH, NO synthase activity is reduced and as a result nitric oxide driven cyclic GMP production via Guanylate Cyclase is also reduced.[76] PDE 5 levels may be increased in PAH and the available PDE 5 inhibitors increase the levels of cyclic GMP. Riociguat enhances the effect of soluble GC and hence increase cyclic GMP.[77] Riociguat has no major side effects except dizziness, urgency micurition, urinary retention, and nasal stuffiness. There is a minor interaction with warfarin and prothrombin levels need to be watched closely in the beginning. Cicletanine has been used as an antihypertensive agent in Europe for the last few years. A phase II trial using 150 mg twice a day or 300 mg daily is currently underway.[78]

PAH can be considered a localized form of neoplasia. There is overexpression of certain growth factors such as platelet-derived growth factor and their receptors in PAH. Activation of these receptors increases tyrosine kinase activity. Well-known chemotherapeutic agents such as imatinib and sorafenib act by attaching themselves to these receptors and decreasing the activity of tyrosine kinase. After satisfactory preliminary results, phase 3 trials (IMPRESS) have been launched using imatinib. Imatinib is being currently used for myelogenous leukemia and gastrointestinal stromal tumors. Sorafenib is a broad-spectrum tyrosine kinase inhibitor, which has been used in the treatment of advanced renal cancer and hepatocellular carcinoma.[79,80]

### Reseeding the lung bed

Whenever there is an injury to the endothelial cells, endothelial progenitor cells (EPCs) originate in the bone marrow and reach the affected areas. It is likely that the initial injury results in widespread endothelial apoptosis and subsequent emergence of apoptosis resistant endothelial cells. Normal EPCs might limit this process by replacing the apoptosis resistant cells. It is entirely possible that EPCs in PAH patients may not work “normally.” Increased levels of bone marrow derived proliferative precursor cells by constantly invading the endothelial vascular bed contribute to excess cell burden in the pulmonary vasculature.[81]

With the development of techniques to isolate cultured EPCs, cell therapy for pulmonary arterial hypertension may become a reality. Lungs microvasculature by acting as sieve will trap the exogenous endothelial cells and altering the internal milieu will restore the normal pulmonary vascular physiology. In animal models transfection of EPCs with eNOS has been shown to increase the levels of NO and provide additional benefit.[82]

EPC therapy has now entered human trials. A randomized study looked at the effects of intravenous autologous EPCs in patients with PAH who were receiving conventional therapy. Significant improvement in 6 minute walk test and hemodynamics were noted 12 weeks after a single bolus of autologous EPCs.[83]

A comprehensive discussion on the management of pulmonary hypertension associated with various connective tissue disease and interstitial lung disease is beyond the scope of this paper. However, it is important to mention the management of pulmonary hypertension in COPD and sleep apnea patients. There is clearly a group of patients in COPD who have significantly higher pulmonary pressure not commensurate with the severity of their disease. These patients need to be treated as aggressively as patients with Idiopathic pulmonary hypertension. In clinical practice it is often very difficult to initiate treatment for pulmonary hypertension based on Echocardiography in a patient with COPD or Interstitial Lung disease. Even after ruling out various other causes of pulmonary hypertension there is currently not enough data to warrant the treatment with specific agents such as sildenafil or Bosentan even though anecdotal case reports of success have been reported. It is generally believed that the pulmonary pressure elevation in sleep apnea patients is minimal and this usually settles down after initiation of Continuous Positive Airway Pressure (CPAP) treatment for these patients. The general rule of thumb in the management of these patients is that when expected improvement is not seen in these patients the physician should be aware of the possibility of the existence of an unrelated pulmonary hypertension and initiate treatment for the same. Even though there is a wealth of information on this topic there is no clear consensus on this issue.

Pulmonary arterial hypertension, until a few years ago was considered a rapidly progressive disorder, ultimately resulting in death. There has been a significant paradigm shift in the treatment of PAH with anticancer medications such as imatinib and sorafenib and more recently with transfusion of autologous EPCs transfected with eNOS to radically replace the pulmonary vascular bed with “normal endothelial cells.” We definitely have entered into an exciting new era in this field with a number of these new medications already being available to treat this condition and the distinct prospect of novel therapies such as tyrosine kinase inhibitors and autologous EPCs.
